# Temperature dependence of electrical characteristics of Pt/GaN Schottky diode fabricated by UHV e-beam evaporation

**DOI:** 10.1186/1556-276X-8-481

**Published:** 2013-11-15

**Authors:** Ashish Kumar, Shamsul Arafin, Markus Christian Amann, Rajendra Singh

**Affiliations:** 1 Department of Physics, Indian Institute of Technology Delhi, New Delhi 110016, India; 2 Walter Schottky Institut, Technische Universität München, Garching 85748, Germany

**Keywords:** Temperature dependence, Pt/GaN Schottky diode, UHV e-beam evaporation, Hall effect, 73.30. + y, 73.40.-c, 79.40. + z, 85.30.Hi

## Abstract

Temperature-dependent electrical characterization of Pt/n-GaN Schottky barrier diodes prepared by ultra high vacuum evaporation has been done. Analysis has been made to determine the origin of the anomalous temperature dependence of the Schottky barrier height, the ideality factor, and the Richardson constant calculated from the *I-V-T* characteristics. Variable-temperature Hall effect measurements have been carried out to understand charge transport at low temperature. The modified activation energy plot from the barrier inhomogeneity model has given the value of 32.2 A/(cm^2^ K^2^) for the Richardson constant A** in the temperature range 200 to 380 K which is close to the known value of 26.4A/(cm^2^ K^2^) for n-type GaN.

## Background

GaN has been the subject of strategic research among all compound semiconductors and has been explored widely and rightly for its various characteristics, like direct wide band gap, high breakdown field, high saturation velocity, and chemical and radiation hardness [1]. The combination of all these properties makes GaN a preferred material for optoelectronics and high-temperature and high-power RF applications. In applications like power rectifier and HEMT, a metal–semiconductor contact with high Schottky barrier height (SBH), high rectification efficiency, and low reverse leakage current is needed [1,2]. Also, the quality of the metal–semiconductor interface is affected by the process steps and deposition vacuum since contamination and oxide layer growth at the interface may result in SBH reduction and high leakage current by inducing local nanoscopic patches of low barrier heights. Werner and Güttler reported that these local patches follow a Gaussian distribution of barrier height and locally control the device characteristics in different temperature regimes of operation [3]. Studies by Tung revealed that this kind of inhomogeneous behavior is observed in all semiconductors and results in overall decreased barrier heights [4]. The contamination level and oxide layer can be minimized by following fabrication steps in a clean room and depositing Schottky metals in ultra high vacuum (UHV). According to the Schottky-Mott model, the Schottky barrier height is dependent on the metal work function and electron affinity of semiconductor *χ* (GaN *χ* = 4.1 eV) [1,5,6]. Metals like Pt, Ni, Pd, and Au which have high work function than GaN make a better choice for gate contact. Pt has a high work function (5.65 eV) that makes it ideal for use as Schottky contacts on n-type GaN, and it is also resistant to oxidation and corrosion [1]. There are only a few reports on Pt/GaN Schottky barrier diodes. The Schottky barrier height of Pt/n-GaN has been reported with a value between 0.89 and 1.27 eV [7-12]. In the present paper, we report an investigation on good-quality Pt/GaN Schottky barrier diodes deposited in ultra high vacuum condition. Temperature-dependent *I-V* characteristics have been measured and analyzed using the barrier inhomogeneity model proposed by Werner and Güttler [3].

## Methods

**Figure 1:**
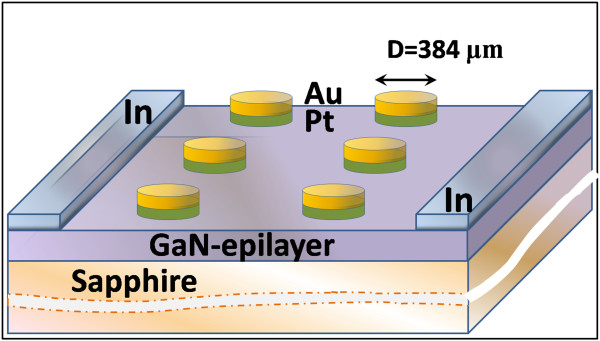
Schematic diagram of the Pt/n-GaN Schottky barrier diode fabricated on n-GaN epitaxial layers.

GaN epitaxial layers used in this study were grown on a c-plane sapphire substrate by metal organic chemical vapor deposition (MOCVD). The GaN epitaxial layers were 3.4 μm thick and unintentionally doped (*N*_D_^*+*^ approximately 3 × 10^16^ cm^-3^ by Hall measurements). For Pt/n-GaN diodes fabricated with indium ohmic contacts on n-GaN epilayers, first the sample was cleaned sequentially with (1) methylpropanol (MP) at around 80°C for 8 min, (2) deionized (DI)water dip, (3) acetone at 50°C for 7 min, (4) isopropanol in ultrasonic bath for 3 min, and again a (5) DI water rinse and dry nitrogen blowing for drying the sample. After that contact, metallization was done by lithography/lift-off techniques. Photoresist (AZ5214), developer (AZ 400 K/H_2_O 1:4), and native oxide layer removal (50% HCl for 1 min, rinse in H_2_O) were applied. Then the sample was immediately transferred to an UHV deposition facility (base pressure in the vacuum chamber was 10^-10^ mbar) for Pt/Au (100/100 nm) Schottky contact deposition. All these steps were carried out in a Class 100 cleanroom facility. Indium (In) ohmic contacts were deposited at two opposite edges by soldering in - second step. The schematic view of the Schottky barrier diodes fabricated in this work is shown in Figure 1. The current–voltage (*I-V*) characteristics of the devices were measured using a programmable Keithley SourceMeter (model 2400, Keithley Instruments, Inc., Cleveland, OH, USA) in the temperature range 100 to 380 K with a temperature step of 40 K in an LN_2_ cryostat. Temperature-dependent Hall and resistivity measurements on GaN epitaxial layer were performed using a variable-temperature Hall setup from Ecopia Corporation, Anyang-si, South Korea (model HMS 5300).

## Results and discussion

**Figure 2:**
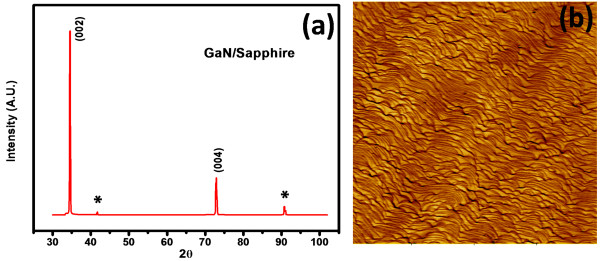
**X-ray diffraction spectrum (a) and AFM image (b) of the GaN epitaxial layer.** The asterisk ‘*’ indicates peaks from sapphire substrate.

Before the fabrication of metal/n-GaN contacts, structural and morphological characterizations of epitaxial layers have been done. The X-ray diffraction pattern of the GaN epitaxial layer using Cu-Kα radiation is shown below in Figure 2a. The X-ray diffraction pattern was taken in bulk mode. The orientation of the epitaxial layer was observed to be along the (002) which confirms the growth of the epitaxial layer along the [0001] direction having a hexagonal (wurtzite) crystal structure. Additional diffraction peaks from (102), (004), and (203) reflection planes of hexagonal GaN were also observed. The sharp diffraction peaks (FWHM value 432 arc sec for (002)) reveal the reasonably good crystalline quality of the GaN epitaxial layer [13]. The lattice constants ‘*a*’ and ‘*c*’ were found to be 0.320 and 0.518 nm, respectively, which matched well with the standard cell parameter values as given in JCPDS card 02–1078. GaN epitaxial layers were also examined under an atomic force microscope (AFM) in the contact mode to measure the topography of the surface. Figure 2b shows the AFM images in a 2D view for the pristine samples. The surface area scanned was 10 × 10 μm^2^. The RMS roughness of the surfaces is around 1 nm for all samples. The result of the AFM measurement shows an overall smooth GaN surfaces. These samples have an average dislocation density value of about 5 × 10^8^ cm^-2^, which is acceptable for GaN epilayers but poor as compared to Si and GaAs epilayers.

**Figure 3:**
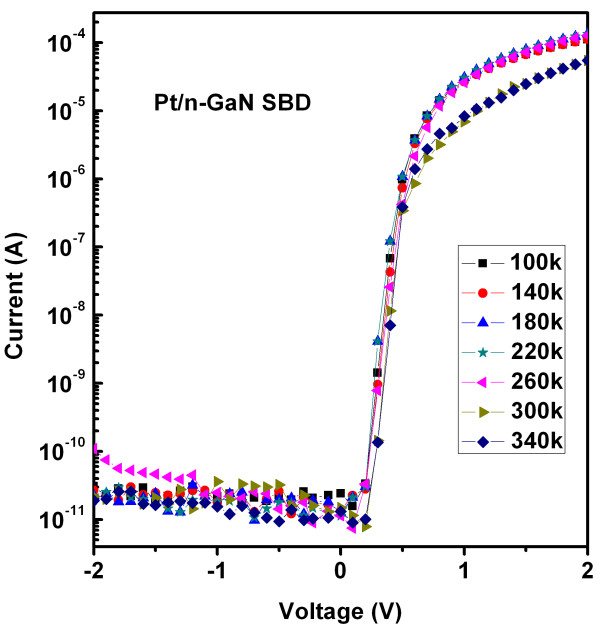
**Semilog forward and reverse *I-V *** **characteristics for Pt/n-GaN Schottky diode at 100 to 340 K.**

Electrical characterization of Schottky barrier devices was carried out in the temperature range of 100 to 340 K measured at a temperature interval of 40 K. Figure 3 shows the experimental semilog forward and reverse bias *I-V* characteristics of the Pt/n-GaN Schottky barrier diodes (SBD). It should be mentioned here that for analysis, we have used diodes with 384-μm diameter and have almost identical electrical properties. The characteristics shown here demonstrate an average trend which was determined for a group of diodes. The current–voltage characteristics of SBD are given by the thermionic emission theory [14,15]. For bias voltage *V* ≥ *3kT*/*q*, the conventional diode equation is

(1) $$I=I_{0}\exp\left(\frac{\text{qV}}{\text{nkT}}\right)$$

(2) $$I_{0}=AA^{**}T^{2}\text{exp}\left(\frac{-q\phi_{\mathrm{ap}}}{\mathrm{kT}}\right).$$

Here, A** is the effective Richardson constant, *ϕ*_ap_ is the apparent or measured barrier height, *n* is the ideality parameter, *A* is the diode area, and the other symbols have their usual meanings. Since image force is a very weak function of applied voltage, it could also be neglected [14-18]. The experimental *I-V* data is plotted as log *I* versus *V* and SBH, and *n* is calculated from the intercept and slope of the linear fit to the linear part of forward characteristics as given by Equations 2 and 3:

(3) $$n=\frac{q}{\text{kT}\;\left(\frac{\mathrm{dV}}{d\ln I}\right)}.$$

**Figure 4:**
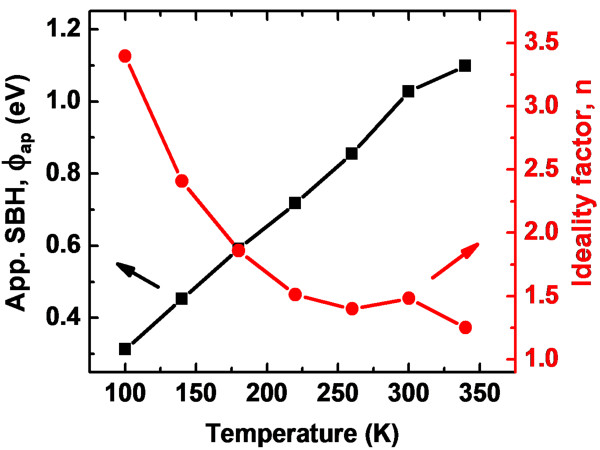
Apparent SBH and ideality factor versus temperature plots for the Pt/n-GaN Schottky diode.

The measured *ϕ*_ap_, *n*, and reverse leakage current (*I*_R_ at -1 V) are listed in Table 1. The Schottky barrier height and the ideality factor of the Pt contact are 1.03 eV and 1.38, respectively. The experimental values of SBH (*ϕ*_ap_) and *n* vary from 1.1 eV and 1.25 (340 K) to 0.31 eV and 3.40 (100 K), respectively. The value of room temperature (300 K) SBH and *n* are 1.03 eV and 1.48, respectively. The measured SBH value of 1.03 eV for the Pt/n-GaN at 300 K is lower than the ideal value of 1.54 eV, calculated according to the Schottky-Mott model. High series resistance was found approximately 10 kΩ at RT, as calculated by the Cheung and Cheung method [19]. The SBH (*ϕ*_ap_) and ideality factor versus temperature plots are given in Figure 4. The SBH decreases and the ideality factor increases with decrease in temperature. Temperature dependence of the measured SBH from the forward bias *I-V* is usually explained in terms of the temperature dependence of the semiconductor band gap. However, in ‘real’ Schottky diodes, it is commonly observed that the temperature coefficient of the SBH differs substantially from the bandgap temperature coefficient and is often of the opposite sign. Such a temperature dependence of both the SBH and ideality factor *n* has often been accredited to current transport mechanisms not following the ideal thermionic emission theory. Various studies have cited different reasons for this nonideal dependence. Werner and Güttler [3] proposed that such dependence originates from Schottky barrier inhomogeneity, which could be due to different interface qualities. The quality of the interface depends on several factors such as surface defect density, surface treatment (cleaning, etching, etc.), deposition processes (evaporation, sputtering, etc.), and local enhancement of electric field which can also yield a local reduction of the SBH [3,16,17,20-22]. This leads to inhomogeneities in the transport current [3,16,17,20-22].

**Table 1:** Calculated Schottky diode parameters for Pt/n-GaN Schottky diodes

**Temperature (K)**	**Ideality factor**	**Apparent SBH (eV)**	**Reverse leakage current (*I***_**R**_**) at** ***V***_**R**_ **= -1 V**
100	0.31	3.40	6 × 10^-11^
140	0.45	2.41	1 × 10^-11^
180	0.59	1.86	4 × 10^-11^
220	0.72	1.51	2 × 10^-12^
260	0.85	1.40	5 × 10^-11^
300	1.03	1.48	5 × 10^-11^
340	1.10	1.25	5 × 10^-11^

The barrier inhomogeneity model assumes a continuous spatial distribution of the local Schottky barrier patches. The shape and position of the ridges in the potential ‘mountains’ depend on bias voltage and cause, therefore, idealities *n* > 1 in *I-V* curves. The total current across a Schottky diode is obtained by integrating the thermionic current expression with an individual SBH and weighted using the Gaussian distribution function across all patches. This approach, however, does not consider the lateral length scale of the inhomogeneity and the pinch-off effect related to the interaction between adjacent regions with different SBHs. The approach points out that the apparent SBH is always lower than the mean value of the barrier distribution and is given with the following expression [3,17,18,23]:

(4) $$\phi_{\mathrm{ap}}=\phi_{\mathrm{bo}}-\frac{q\sigma_{\mathrm{so}}^{2}}{2\mathrm{kT}},$$

where *ϕ*_ap_ is the apparent SBH measured from the forward bias *I-V* characteristics and *σ*_so_ is the zero-bias standard deviation of the SBH distribution and a measure of the barrier homogeneity. The temperature dependence of *σ*_so_ is usually small and can be neglected. Thus, SBH has a Gaussian distribution with the zero-bias mean SBH, *ϕ*_bo_. The variation in ideality factor *n* with temperature in the model is given by [3,17,24]

(5) $$\left(n^{-1}-1\right)=-\rho_{2}-\frac{q\rho_{3}}{2\mathrm{kT}}.$$

The voltage-independent ideality factor *n* requires a linear increase in *ϕ*_b_(*V*, *T*) with the bias. This is only possible if the mean SBH *ϕ*_b_ as well as the square of the standard deviation *σ*^2^ varies linearly with the bias [3,17,18,24]:

(6) $$\Delta \phi_{b}\left(V,T\right)=\phi_{b}\left(V,T\right)-\phi_{b}\left(0,T\right)=\rho_{2}V$$

(7) $$\Delta \sigma^{2}\left(V\right)=\sigma^{2}\left(V\right)-\sigma^{2}\left(0\right)=\rho_{3}V.$$

**Figure 5:**
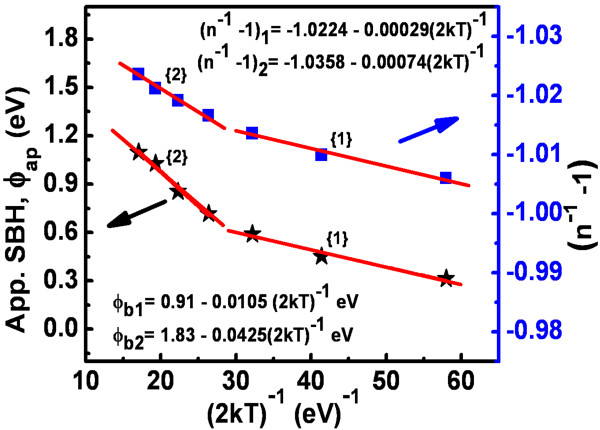
**Zero-bias apparent barrier height (stars) and ideality factor function (*n***^**-1**^ **- 1) versus 1/(*2kT*) (filled boxes) curves.**

As can be seen from Equations 6 and 7, *ρ*_2_ is the voltage coefficient of the mean SBH, and *ρ*_3_ is the voltage coefficient of the standard deviation. According to Equation 5, a plot of (*n*^-1^- 1) against 1/*T* should give a straight line with the slope and *y*-axis intercept related to the voltage coefficients *ρ*_2_ and *ρ*_3_, respectively. The value of *ρ*_3_ indicates that the distribution of the SBH becomes more homogeneous with voltage increase. A linear *ϕ*_ap_ versus 1/*T* curve means that the plot obeys the barrier inhomogeneity model. The experimental (*n*^-1^- 1) and *ϕ*_ap_ versus 1/*T* plots in Figure 5 correspond to two lines instead of a single straight line with transition occurring at 200 K. The values of *ρ*_2_ obtained from the intercepts of the experimental (*n*^-1^ - 1) versus 1/*T* plot are shown in Figure 5. The intercept and slope of the straight line have given two sets of values of *ϕ*_bo_ and *σ*_so_ in the temperature range of 100 to 180 K and in the temperature range of 220 to 340 K, respectively. Our results are similar to the results obtained for Pd/n-GaN and Pt/n-GaN in the temperature range of 80 to 400 K [25].

Further, the conventional saturation current expression can be written for the activation energy plot or Richardson plot by rewriting Equation 2 as follows:

(8) $$\text{ln}\;\left(\frac{I_{0}}{T^{2}}\right)=\ln\;\left(AA^{**}\right)-\frac{q\phi_{\mathrm{ap}}}{\mathrm{nkT}}.$$

The conventional activation energy ln(*I*_0_/*T*^2^) versus 1/*T* plot should be linear in ideal case and gives A** and SBH as intercept and slope calculations based on the TE current mechanism. For inhomogeneous diodes, this is not true. Therefore, a modified activation energy expression according to the Gaussian distribution of the SBHs can be rewritten by incorporating Equations 4 and 5 in Equation 8:

(9) $$\ln\;\left(\frac{I_{0}}{T^{2}}\right)-\frac{q^{2}\sigma_{\mathrm{so}}^{2}}{2k^{2}T^{2}}=\ln\;\left(AA^{**}\right)-\frac{q\phi_{\mathrm{bo}}}{\mathrm{nkT}}.$$

**Figure 6:**
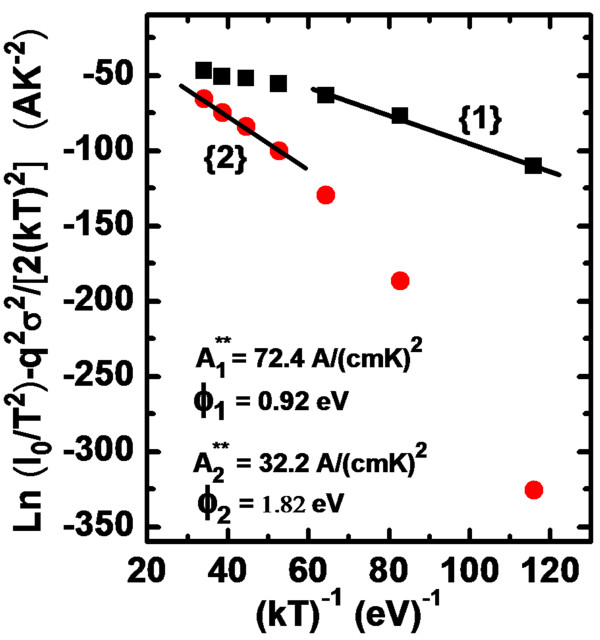
**Modified Richardson plot, [ln(*I***_**0**_**/*T***^**2**^**) -** ***q***^**2**^***σ***_**so**_^**2**^**/2*k***^**2**^***T***^**2**^**] versus 1/*T*, for the Pt/n-GaN Schottky diode.**

Using the experimental *I*_0_ data, the modified activation energy plot or Richardson plot ($\left[\ln\left(I_{0}/T^{2}\right)-q^{2}\sigma_{\mathrm{so}}^{2}/2k^{2}T^{2}\right]$ versus 1/*T*) can be obtained according to Equation 9. This plot should give a straight line with a slope directly yielding the mean *ϕ*_bo_ and the intercept (=ln(AA**)) at the ordinate determining A** for a given diode area *A*. The theoretical value of A** can be calculated using A** = 4πm*qk^2^/*h*^3^, where *h* is Planck’s constant. For n-type GaN, m* = 0.22*m*_o_ is the effective electron mass for GaN and the value of A** is determined to be 26.4 A/(cm^2^K^2^). Zhou et al. [21] also reported that the value of A** determined by a modified Richardson plot in the GaN material is close to the theoretical value. The $\left[\ln\left(I_{0}/T^{2}\right)-q^{2}\sigma_{\mathrm{so}}^{2}/2k^{2}T^{2}\right]$ values were calculated using both values of *σ*_so_ obtained for the temperature ranges of 100 to 220 and 220 to 340 K. Thus, in Figure 6, the circles represent the plot calculated with *σ*_so_ = 90 mV (straight line 1) in the temperature range of 100 to 200 K, and the squares represent the plot calculated with *σ*_so_ = 176 mV (straight line 2) in the temperature range of 200 to 380 K. The best linear fits to the modified experimental data are depicted by solid lines in Figure 6 which represent the true activation energy plots in respective temperature ranges. The calculations have yielded zero-bias mean SBH *ϕ*_bo_ of 0.92 eV (in the range of 100 to 220 K) and 1.82 eV (in the range of 220 to 340 K). In Figure 6, the intercepts at the ordinate give the Richardson constant A** as 72.4 A/(cm^2^K^2^) (in the range of 100 to 220 K) and 32.2A/(cm^2^K^2^) (in the range of 220 to 340 K) without using the temperature coefficient of the SBHs. This value of the Richardson coefficient at room temperature is close to the theoretical value 26.4A/(cm^2^K^2^) [14,16-20,23]. It can be pointed out that although a barrier inhomogeneity is visible in Pt/GaN diodes, But highlighting feature of these diodes, is high Schottky barrier height observed. The quality of the metal–semiconductor interface is affected by the process steps and deposition vacuum since contamination and oxide layer growth at the interface may result in SBH reduction and high leakage current by inducing local nanoscopic patches of low barrier heights. Studies by Iucolano et al. revealed that this kind of inhomogeneous behavior is observed in all semiconductors and results in overall decreased barrier heights [10]. The contamination level and oxide layer can be minimized by following fabrication steps in a clean room and depositing Schottky metals in UHV. By selecting high work function metal Pt, a high gate potential can be achieved. These kinds of high barrier heights are suitable for many high-power and switching applications. The reverse characteristics of these devices are also quite good as compared to those of other Schottky metal combinations. Very low reverse leakage current and high breakdown voltages are good for high-power applications where losses should be low. A high rectifying ratio is desired for switching applications. These diodes are better in terms of observed Schottky barrier height and reverse characteristics.

**Figure 7:**
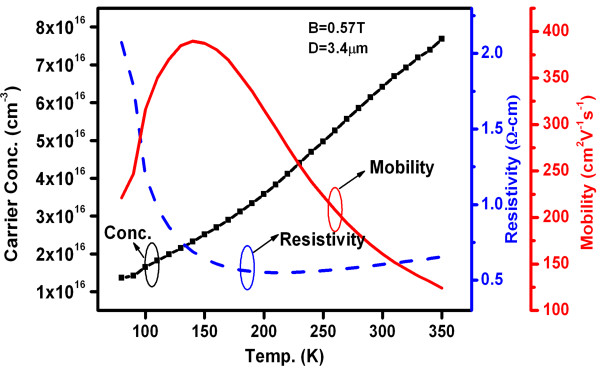
**Carrier concentration (*N***_**D**_^***+***^**), resistivity (*ρ*), and mobility (*μ*) as a function of temperature for n-GaN.**

To physically understand the origin of this barrier inhomogeneity in two different temperature regimes and to comprehend the current transport at low temperatures, we performed variable-temperature Hall measurements. In Figure 7, the *N*_D_^*+*^ (carrier concentration) values measured from Hall measurements are shown for the temperature range of 80 to 350 K for n-type GaN samples. It is well known that *N*_C_ for n-type GaN samples is NC=22πm*kT/h232_,_ where m* is the electron effective mass (m* = 0.22*m*_o_ for n-GaN, where *m*_o_ is the free electron mass) and *h* is Planck's constant. The *N*_C_ values in the temperature range of 100 to 350 K are also calculated (not shown here). As can be seen in Figure 7, the *N*_D_^*+*^ of the n-type GaN increases with an increase in temperature. The ratio *N*_C_/*N*_D_^*+*^ at 350 K is greater than *N*_C_/*N*_D_^*+*^ at 100 K. Since EC-EF=kT×lnNCND+ (where symbols have usual meanings), this leads to reduction in *E*_C_ - *E*_F_ in the n-type GaN bulk with decreasing temperature from 350 to 100 K. The reduction in *E*_C_ - *E*_F_ might cause a relatively higher value of built-in potential that can lead to the fact that this SBD will transport less current as compared to SBD with comparatively less built-in potential [26]. Also, the decrease in *E*_C_ - *E*_F_ at low temperature may also lead to addition of currents other than thermionic current, such as thermionic field emission and field emission currents [26]. This also explains the increase in ideality factor (*n*) at low temperatures. Thus, inhomogeneous Schottky barrier patches might also have varied built-in potential at lower temperature resulting in two portions of barrier inhomogeneity dependency in Figures 5 and 6.

## Conclusions

In conclusion, a detailed electrical analysis of the Pt/n-GaN Schottky contacts prepared by evaporation has been made to determine the origin of the anomalous temperature dependence of the SBH, the ideality factor, and the Richardson constant calculated from the *I-V-T* characteristics. The variable-temperature Hall experiments have given an insight into the origin of barrier inhomogeneity observed commonly in n-GaN-based Schottky barrier diodes. The temperature dependence of the experimental values of SBH of the Pt/n-GaN has been described by two Gaussian distributions in the temperature range of 100 to 340 K. The modified activation energy plot from the barrier inhomogeneity model has given the value of 32.2 A/(cm^2^ K^2^) for the Richardson constant A** in the temperature range 200 to 380 K which is close to the known value of 26.4 A/(cm^2^ K^2^) for n-type GaN.

## Competing interests

The authors declare that they have no competing interests.

## Authors’ contributions

AK carried out the research, drafted this manuscript. SA contributed in device fabrication. MCA is the research collaborator who provided experimental facilities. RS is PhD supervisor of AK. The manuscript was sent to all contributors. All authors read and approved the final manuscript.
